# Associations of Environmental Pollutant Mixtures and Red Blood Cell Folate Concentrations: A Mixture Analysis of the U.S. Adult Population Based on NHANES Data, 2007–2016

**DOI:** 10.3390/toxics13030200

**Published:** 2025-03-11

**Authors:** Michael Mascari, Katherine Reeves, Raji Balasubramanian, Zhenhua Liu, Nasser Laouali, Youssef Oulhote

**Affiliations:** 1 Department of Epidemiology and Biostatistics, School of Public Health & Health Sciences, University of Massachusetts Amherst, Amherst, MA 01003, USA; mmascari@umass.edu (M.M.); kwreeves@umass.edu (K.R.); rbalasub@schoolph.umass.edu (R.B.); 2 Department of Nutrition, School of Public Health & Health Sciences, University of Massachusetts Amherst, Amherst, MA 01003, USA; zliu@nutrition.umass.edu; 3 Faculty of Medical Sciences, Mohammed VI Polytechnic University, Benguerir 43150, Morocco; nasser.laouali@um6p.ma; 4 Department of Environmental Medicine and Climate Science, Icahn School of Medicine at Mount Sinai, New York, NY 10029, USA

**Keywords:** folate, pollutants, NHANES, mixtures, metals, PFAS, EDCs, PAHs

## Abstract

Background: Folate is critical for many physiological processes, and low folate levels have been associated with a wide range of health outcomes, including chronic diseases and developmental outcomes. Many environmental chemicals are suspected to contribute to the etiology of health outcomes related to folate deficiency. However, little is known about how these pollutants influence folate levels as potential mechanistic pathways. Objective: To investigate the individual and joint associations between a mixture of 39 pollutants and red blood cell (RBC) folate concentrations in the U.S. population. Methods: We used available data on 27,938 participants, aged 18–80 from the U.S. National Health and Nutrition Examination survey (2007–2016), with available RBC folate concentrations and 39 environmental pollutants’ concentrations. We estimated covariate-adjusted independent and joint associations between environmental pollutants and RBC folate, and compared evidence from two complimentary mixture approaches: exposome-wide association study (ExWAS) and quantile-based g computation (Q-gcomp). Results: In the ExWAS analysis, 12 environmental chemicals, including metals (cadmium, arsenic, lead, and mercury), perfluoroalkyl substances, phthalates, phenols and parabens, and polycyclic aromatic hydrocarbons, were inversely associated with RBC folate, whereas four environmental pollutants, including metals (manganese and selenium) and two phthalate metabolites, were positively associated with RBC folate. Q-gcomp showed convergent results with the ExWAS analysis; a quartile increase in the metal and PFAS mixtures was significantly associated with a decrease of −38.4 ng/mL (95%CI: −52.3, −24.4) and −48.9 ng/mL (95%CI: −57.6, −39.6) in RBC folate concentrations, respectively. Conclusion: The present study shows that higher exposure to PFASs, metals, and PAHs are associated with lower RBC folate concentrations. However, given the cross-sectional design, we cannot make inferences about the directionality of the observed associations.

## 1. Introduction

Folate is the generic term for naturally occurring folate forms in foods, and folic acid, also known as vitamin B_9_ or Vitamin M, is the synthetic form of folate used for dietary supplements and fortified foods. Folate is vital in humans for several metabolic reactions involved in the formation and transfer of methyl groups. These metabolic reactions in humans include the following: biosynthesis of purines and thymidine, amino acid homeostasis of glycine, serine, and methionine, epigenetic maintenance, homocysteine remethylation, hematopoiesis, and immune responses [1,2]. Folate deficiency has been linked to anemia [1,2] and hyperhomocystinemia [3], which has been associated with increased cardiovascular, cerebrovascular, and thromboembolic diseases. There is also epidemiologic evidence to sμggest folate is inversely associated with various cancers including lung, oropharynx, esophagus, stomach, colorectal, pancreas, cervix, ovary, prostate, and breast cancers, as well as leukemia [4,5,6]. Additionally, low folate levels have been associated with a range of developmental outcomes, including autism, neural tube defects, neurodevelopment, still birth, preterm birth, and recurrent pregnancy loss [7,8,9,10,11,12,13,14,15,16]. A recent NHANES biomonitoring study sμggested that, althoμgh blood folate concentrations in the US population have not decreased recently, the prevalence of folate insufficiency is about 20% in the US population [7].

There are both modifiable and non-modifiable factors associated with low folate levels. Established modifiable factors affecting folate levels include intake of certain medications, alcohol use, smoking, and dietary intake of folic acid supplements [17,18,19,20]. On the other hand, single nucleotide polymorphisms in certain genes are genetic, non-modifiable risk factors that affect folate levels. These include SNPs in 5,10-methylenetertahydrofolate, and other genes responsible for the metabolism of vitamins, as well as mutations in solute carrier family 46 member 1 (SLC46A1), a gene responsible for folate transport [21].

Recently, exposure to environmental chemicals has been sμggested as a potential modifiable risk factor for low folate levels. Environmental chemicals refer to chemicals that are present in air, water, food, soil, dust, or other environmental media such as consumer products [15]. These chemicals are often ubiquitous in the environment, and some may persist for several years. Many of these chemicals had limited testing for their effects on human health and even less is known about the combined exposure to many environmental chemicals [22].

The objective of this study is to investigate the associations between individual chemicals, chemical mixtures, and red blood cell (RBC) folate levels among the U.S. adult population using available data from the National Health and Nutrition Examination Survey (NHANES; 2007–2016). We compared evidence from two complimentary statistical approaches developed to examine chemical mixtures: exposome-wide association study (ExWAS) and a quantile-based g computation (Q-gcomp).

## 2. Methods

### 2.1. Population and Data Collection

The National Health and Nutrition Examination Survey (NHANES) is a cross-sectional, nationwide study designed for the assessment of the health and nutritional status of noninstitutionalized adults and children in the United States, conducted by the Centers for Disease Control and Prevention (CDC). Questionnaires were administered by study staff at an in-home visit and biological specimens were collected at mobile examination centers (MEC) [23].

This analysis included 27,938 NHANES participants aged 18 years and older. All measures of environmental chemicals and RBC folate concentrations were conducted as part of the NHANES program at the CDC, and laboratory methods for blood and urine samples have been described thoroμghly at: https://wwwn.cdc.gov/nchs/nhanes/search/datapage.aspx?Component=Laboratory (accessed 1 December 2018). All the data used in this analysis are available at the CDC website and were extracted from the following link: https://wwwn.cdc.gov/nchs/nhanes/Default.aspx (accessed 1 December 2018).

### 2.2. RBC Folate Measurement

RBC folate was processed, stored, and shipped to the Division of Laboratory Sciences, National Center for Environmental Health, and Centers for Disease Control and Prevention for analysis. RBC folate concentrations were measured using a microbiological assay, which was described in detail elsewhere [24].

### 2.3. Assessment of Environmental Exposure Biomarkers

We included six environmental chemical families in this analysis: phthalates, heavy metals, per- and polyfluoroalkyl substances (PFASs), phenols and parabens, polyaromatic hydrocarbons (PAHs), and cotinine. These environmental chemical families included 39 environmental chemical biomarkers and metabolites. Phthalates, arsenic, phenols and parabens, and PAHs were measured in urine samples collected at mobile examination centers (MECs) [23]. PFASs and cotinine were measured in serum samples collected at MECs, whereas metals were measured in whole blood samples.

### 2.4. Phthalates (ng/mL)

Measures of urinary phthalate metabolites were performed in a subsample comprising one-third of the participants in all the included cycles. There were 11 phthalate metabolites included in this study: Mono (carboxyisoctyl) phthalate (MCOP), Mono-2-ethyl-5-carboxypentyl phthalate (MECPP), Mono-n-butyl phthalate (MBP), Mono-(3-carboxypropyl) phthalate (MCPP), Mono-ethyl phthalate (MEP), Mono-(2-ethyl-5-hydroxyhexyl) phthalate (MEHHP), Mono-(2-ethyl)-hexyl phthalate (MEHP), Mono-isobutyl phthalate (MiBP), Mono-isononyl phthalate (MNP), Mono-(2-ethyl-5-oxohexyl) phthalate (MEOHP), and Mono-benzyl phthalate (MBzP). Urine samples for the quantification of phthalate metabolites were stored at −20 °C until they arrived at the National Center for Environmental Health for testing. They were quantified using high performance liquid chromatography–electrospray ionization–tandem mass spectrometry (HPLC-ESI-MS/MS). The percentage coefficient of variation (CV) for phthalates ranged from 1.5% to 18.9%. There was a range in limits of detection (MCOP: 0.2–0.7 ng/mL; MECPP: 0.2–0.5 ng/mL; MBP: 0.4–0.6 ng/mL; MCPP: 0.2–0.4 ng/mL; MEP: 0.462–1.2 ng/mL; MEHHP: 0.2–0.7 ng/mL; MEHP: 0.5–1.1 ng/mL; MiBP: 0.2–0.8 ng/mL; MNP: 0.5–1.232 ng/mL; MEOHP: 0.2–0.6 ng/mL; MBzP: 0.2–0.3 ng/mL) based on the NHANES cycle.

### 2.5. Heavy Metals

Blood metals were measured in all participants in all cycles, whereas urine metals were determined only in subsamples of participants in all cycles. Heavy metals measured in blood included cadmium (μg/L), lead (μg/dL), manganese (μg/L), mercury (μg/L), and selenium (μg/L). Blood samples for the quantification of heavy metals were stored at −30 °C until they arrived at the National Center for Environmental Health for testing. Samples of total urinary arsenic samples (μg/L) were stored at −30 °C until they arrived at the National Center for Environmental Health for testing. They were quantified using inductively coupled plasma-mass spectrometry (ICP-MS). The percent CV for metals ranged from 1.2% to 11.3%. There was a range in the limits of detection (cadmium: 0.10–0.28 μg/L; lead: 0.07–0.37 μg/dL; manganese: 0.99–1.06 μg/dL; mercury: 0.16–0.325 μg/dL; selenium: 24.48–30.0 μg/dL; arsenic: 0.26–1.25 μg/dL) based on the NHANES cycle.

### 2.6. PFAS (ng/mL)

Measures of serum PFAS compounds were performed in a one third subsample of participants in all the included cycles. There were 7 PFAS compounds included in this study: Perfluorooctanoic acid (PFOA), Perfluorooctane sulfonic acid (PFOS), Perfluorononanoic acid (PFNA), Perfluoroundecanoic acid (PFUA), Perfluorohexane sulfonic acid (PFHxS), Perfluorodecanoic acid (PFDeA), and 2-(N-methylperfluoroctanesulfonamido)acetic acid (Me-PFOSA-AcOH). Serum samples for the quantification of PFASs were stored at −30 °C until they arrived at the National Center for Environmental Health for testing. They were quantified using solid phase extraction coupled with high performance liquid chromatography–turbo ion spray ionization–tandem mass spectrometry (on-line SPE-HPLC-TIS-MS/MS). The percent CV for PFASs ranged from 8.6% to 12.8%. There was a range of the limits of detection (PFDeA: 0.1–0.2 ng/mL; Me-PFOSA-AcOH: 0.09–0.2 ng/mL; PFNA: 0.08–0.1 ng/mL; PFUA: 0.1–0.2 ng/mL; PFOS: 0.1–0.2 ng/mL) based on NHANES cycle. The limit of detection for PFHxS and PFOA was 0.1 ng/mL for all NHANES cycles.

### 2.7. Phenols and Parabens (ng/mL)

Measures of urinary phenols and parabens were performed in a one third subsample of participants in all the included cycles. There were two phenols and 3 parabens measured in this study: bisphenol A (BPA), triclosan, methyl paraben (MBP), butyl paraben (BPB), and propyl paraben (PBP). Urine samples for the quantification of phenols and parabens were stored at −20 °C until they arrived at the National Center for Environmental Health for testing. They were quantified using on-line solid phase extraction coupled with high performance liquid chromatography and tandem mass spectrometry (on-line SPE-HPLC-MS/MS). The percent CV for phenols and parabens ranged from 2.3% to 13.5%. There was a range of the limits of detection (BPA: 0.2–0.4 ng/mL; Triclosan: 1.7–02.3 ng/mL; butyl paraben: 0.1–0.2 ng/mL; propyl paraben: 0.1–0.2 ng/mL) based on NHANES cycle. The limit of detection for methyl paraben was 1.0 ng/mL for all NHANES cycles.

### 2.8. PAH (ng/L)

Measures of urinary phthalate metabolites were performed in a subsample of one third of the participants in four cycles (2007–2014) and were not performed in the last cycle (2015–2016). There were 10 PAHs measured in this study: 1-hydroxynaphthalene, 2-hydroxynaphthalene, 3-hydroxyfluorene, 2-hydroxyfluorene, 3-hydroxyphenanthrene, 1-hydroxyphenanthrene, 2-hydroxyphenanthrene, 1-hydroxypyrene, 9-hydroxyfluorene, and 4-phenanthrene. Urine samples for the quantification of PAH were stored at −20 °C until they arrived at the National Center for Environmental Health for testing. They were quantified using on-line SPE-HPLC-MS/MS. The percent CV for PAHs ranged from 2.1% to 13%. There was a range of the limits of detection (1-hydroxynaphthalene: 44.0–60.0 ng/L; 2-hydroxynaphthalene: 42.0–90.0 ng/L; 3-hydroxyfluorene: 4.95–10.0 ng/L; 2-hydroxyfluorene: 8.0–10.041 ng/L; 3-hydroxyphenanthrene: 4.95–10.0 ng/L; 1-hydroxyphenanthrene: 7.778–10.0 ng/L; 2-hydroxyphenanthrene: 4.95–10.0 ng/L; 1-hydroxypyrene: 4.95–70.0 ng/L; 9-hydroxyfluorene: 10.0–18.243 ng/L) based on the NHANES cycle.

### 2.9. Cotinine (ng/mL)

Samples of serum cotinine were stored at −20 °C until they arrived at the National Center for Environmental Health for testing. Cotinine was measured by isotope-dilution high-performance liquid chromatography/atmospheric pressure chemical ionization tandem mass spectrometric (ID HPLC-ACPI MS/MS) method. The percent CV for cotinine ranged from 4% to 40.6%. The limit of detection for serum cotinine was 0.015 ng/mL for all NHANES cycles.

## 3. Covariates

All data on covariates was extracted from the CDC/NHANES website. We identified potential confounders that were associated with environmental chemicals and RBC folate from prior research. Questionnaire data were collected for participants’ sex, age, race, education, and family poverty-to-income ratio (FPR: family’s income divided by the poverty level threshold for the family size and the survey year; a FPR of 1 indicates a family income at 100% of the federal poverty level) at the in-home visit. Urinary creatinine (mg/dL) was assessed from laboratory data and log_2_-transformed for analyses to address skewness and limit the influence of outliers. The healthy eating index (HEI) was also calculated from 24 h dietary recall interviews at the MECs, and was included as a proxy for dietary patterns that may influence both chemical concentrations and RBC folate. The HEI measures overall diet quality based on adherence to the Dietary Guidelines for Americans and is validated in the U.S. population. It consists of adequacy components (total fruits, whole fruits, vegetables, greens/beans, whole grains, dairy, protein foods, seafood/plant proteins, fatty acids) and moderation components (refined grains, sodium, added sμgars, saturated fats). The 100-point scale assigns a higher score for better quality of overall diet. Finally, NHANES cycle was also included in all models to account for secular trends in both RBC folate levels and environmental chemical concentrations. Final models therefore included the following confounders: age, sex, race, education, FPR, urinary creatinine, HEI, and NHANES cycle.

## 4. Statistical Methods

We described distributions of adult sociodemographic characteristics using frequencies and percentages for categorical variables and mean with an accompanying standard deviation for continuous variables. The sample size, percent below the limit of detection (LOD), geometric mean, geometric standard error, interquartile range, and maximum values for each chemical, as well as RBC folate were calculated and presented in descriptive analyses. Environmental chemical concentrations were log_2_-transformed and the influence of outliers was limited. Additionally, values below the limit of detection were replaced by the limit of detection divided by the square root of 2.

We conducted descriptive analyses to compare RBC folate concentrations by adult sociodemographic characteristics using Student’s *t* test or One-way Analysis of Variance (ANOVA) (with Tukey’s test for each category) depending on the number of categories for each sociodemographic characteristic. We calculated the Pearson correlation coefficients for the correlations between different chemicals and between chemicals and RBC folate.

To investigate the individual and joint associations between chemicals and RBC folate, we used two complimentary methods that can consider multiple correlated exposures in the context of environmental health studies: exposome-wide association study (ExWAS) and quantile-based g computation (Q-gcomp).

ExWAS addresses potential issues with type 1 error rate due to multiple comparisons; this method corrects for multiplicity by performing traditional linear regression and applying a threshold for effective tests (TEF) for significance testing [25,26]. The TEF is calculated based on the number of exposures and applies a Bonferroni correction based on the number of exposures.

As a complementary analysis, we used Q-gcomp to investigate the joint associations of multiple chemicals with RBC folate. This method does not assume linearity or additivity, and it does not assume that all components of the mixture have the same direction of effect [27]. We ran Q-gcomp analyses separately for the different families of pollutants, metals, PFASs, phthalates, PAHs, and phenols and parabens.

Because each NHANES cycle contains different amounts of missing data for each chemical, and not all chemicals were measured for all NHANES cycles, our dataset contained missing values for chemicals during the NHANES cycles, as they were not collected or because some chemical families, such as phthalates, phenols and parabens, PFASs, and PAHs were only measured in a subset of participants. To assess the impact of missing values on our study, we conducted a sensitivity analysis using multivariable imputation by chained equations (MICE). MICE is an imputation method that creates multiple imputed datasets to generate more accurate standard error values when using simulated datasets [28]. Based on previous research and imputation guidelines, we generated 70 imputed datasets [28]. We used the Rubin’s rule to combine the results from the 70 imputed datasets for the ExWAS and Q-gcomp analyses [29]. All statistical analyses were conducted using R version 3.5.2. (R Core Team, Vienna, Austria, 2018), and SAS version 9.4 (SAS Institute, Inc, Cary, NC, USA).

## 5. Results

NHANES participants in our sample were predominantly male (48.7%), non-Hispanic white (41.5%), had some college or an associate in arts degree (28.90%), and had not smoked in the past 30 days (79.5%), (Table 1). The geometric mean urinary creatinine concentration was 98.94 (GSD: 2.09) mg/dL, and the average FPR was 2.44 (SD: 1.63). The geometric mean RBC folate concentration was 515.7 ng/mL (geometric standard deviation (GSD) = 243.7 ng/mL). The mean age was 48 (SD: 18.48) years old. RBC folate concentrations were higher among females, participants aged 65 years and older, non-Hispanic whites, participants that had at least a college degree, those who have not smoked in the past 30 days, participants in the highest family income-to-poverty ratio, participants in the lowest quartile of urinary creatinine values, and in the highest quartile of the HEI (Table 1). Geometric mean concentrations and the distribution of phthalates, metals, PFASs, PAHs, and phenols and parabens, are displayed in Table 2.

**Figure 1:**
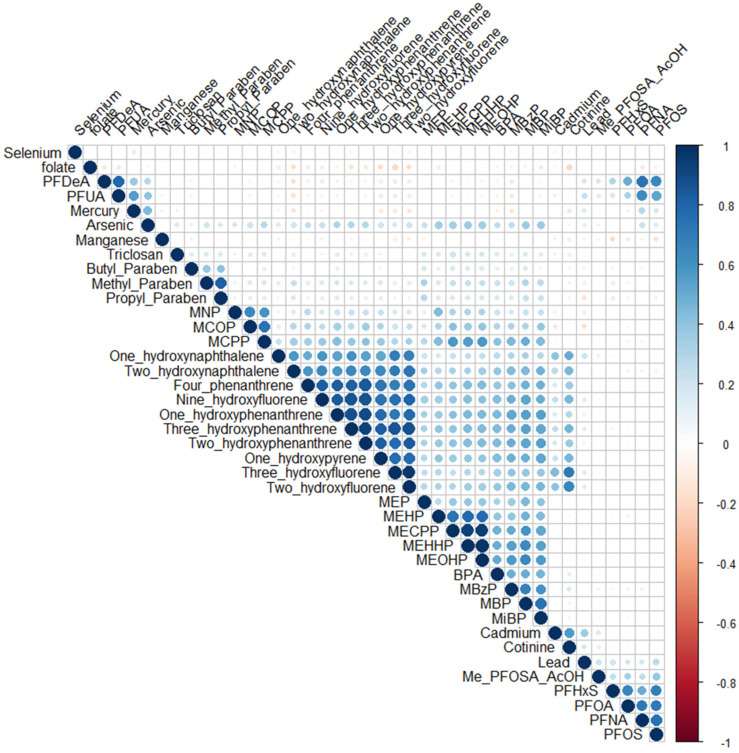
Pearson correlation heat map of included environmental pollutants and folate. Red circles indicate negative correlations and blue circles indicate positive correlations.

Within and between family chemical correlations varied between 0.10 and 0.98 for phthalates, −0.07 and 0.56 for metals, −0.19 and 0.78 for PFASs, 0.28 and 0.96 for PAHs, and −0.11 and 0.82 for phenols and parabens (Figure 1).

### 5.1. ExWAS Analysis

**Figure 2:**
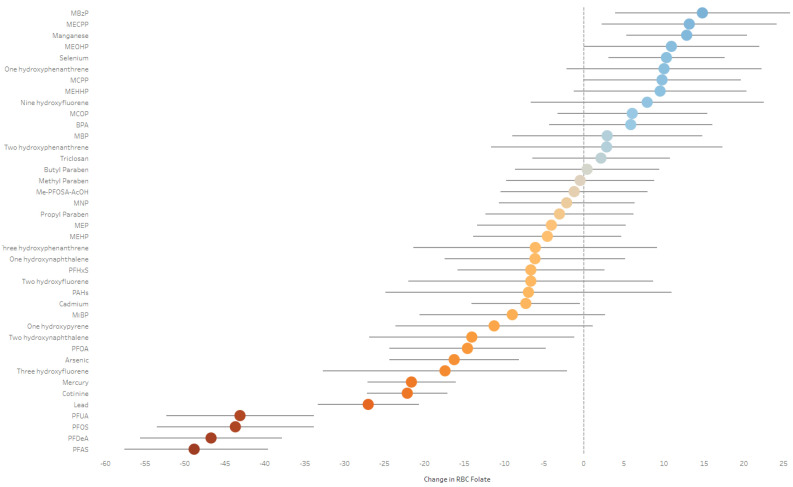
Estimates and 95% CIs for the associations between environmental chemical biomarkers and RBC folate using exposome-wide association study (ExWAS) analysis from the NHANES database from 2007 to 2016 (TEF = 2.01 × 10^−3^). Models were adjusted for age, race, sex, family income-to-poverty ratio, education, NHANES cycle, and HEI. Additional adjustments for urinary creatinine were made for models with chemicals collected in urine. Estimates represent the change in RBC folate concentrations for each doubling in chemical concentrations. Blue illustrates a positive estimate and red illustrates a negative estimate.

For the overall ExWAS analysis, the corrected *p* value was 2.01 × 10^−3^. After adjusting for sex, age, race, education, smoking status, FPR, urinary creatinine, HEI, and NHANES cycle, 12 environmental chemicals, including metals, PFASs, and PAHs, were significantly inversely associated with RBC folate, and four environmental chemicals, including metals (manganese, selenium), and MBzP and MECPP phthalates, were significantly positively associated with RBC folate (Figure 2). For instance, a doubling of PFNA (β = −48.3; 95%CI: −57.4, −39.1), PFDeA (β = −46.7; 95%CI: −55.6, −37.8), PFOS (β = −43.7; 95%CI: −53.5, −33.9), PFUA (β = −43.1; 95%CI: −52.4, −33.9), lead (β = −27.0; 95%CI: −33.4, −20.7), cotinine (β = −22.1; 95%CI: −27.2, −17.1), mercury (β = −21.6; 95%CI: −27.2, −16.1), three hydroxyfluorene (β = −17.4; 95%CI: −32.7, −2.1), arsenic (β = −16.3; 95%CI: −24.4, −8.1), PFOA (β = −14.6; 95%CI: −24.4, −4.8), two hydroxy naphthalene (β = −14.1; 95%CI: −26.9, −1.2), and cadmium (β = −7.3; 95%CI: −14.1, −0.5) were associated with a statistically significant decrease in RBC folate after adjustment. A doubling of MBzP (β = 14.8; 95%CI: 3.9, 25.8), MECPP (β = 13.2; 95%CI: 2.2, 24.1), manganese (β = 12.9; 95%CI: 5.3, 20.4), and selenium (β = 10.3; 95%CI: 3.0, 17.6) were associated with a statistically significant increase in RBC folate after adjustment. No other chemicals were statistically significantly associated with a change in RBC folate after adjustment.

### 5.2. Q-Gcomp Analysis

**Figure 3:**
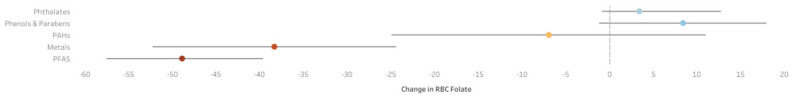
Estimates and 95% CIs for the associations between mixtures of environmental chemical biomarkers and RBC folate using quantile g computation (Q-gcomp) analysis from the NHANES database from 2007 to 2016. Models were adjusted for age, race, family income-to-poverty ratio, education, NHANES cycle, smoking status, and HEI. Additional adjustments for urinary creatinine were made for models with chemicals collected in urine. Estimates represent the associations between one quartile increases in an exposure family and changes in RBC folate concentrations.

One quartile increases in the metal (β = −38.36; 95%CI −52.31, −24.42) and PFAS (β = −48.86; 95%CI: −57.56, −39.61) mixtures were significantly associated with a decrease in RBC folate after adjustment. No other chemical families were statistically significantly associated with a change in RBC folate after adjustment (Figure 3).

**Figure 4:**
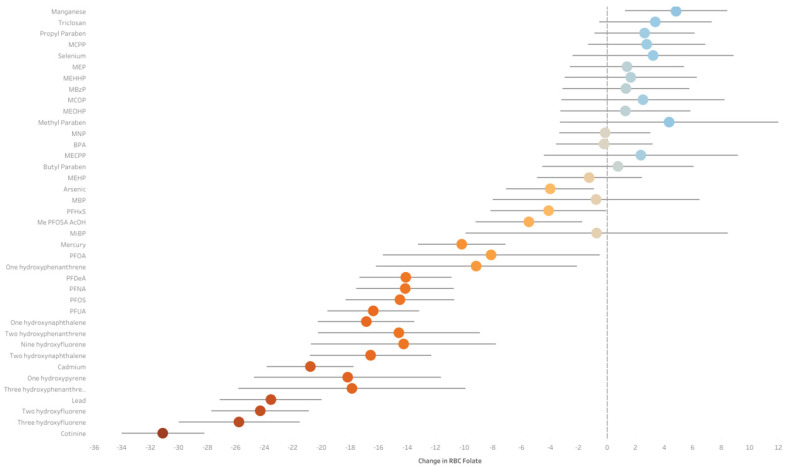
Pooled estimates and 95% CIs for the associations between environmental chemical biomarkers and RBC folate using exposome-wide association study (ExWAS) analysis from the NHANES database from 2007 to 2016 using 70 imputed datasets (TEF = 2.01 × 10^−3^). Models were adjusted for age, race, sex, family to income poverty ratio, education, NHANES cycle, and HEI. Additional adjustments for urinary creatinine were made for models with chemicals collected in urine. Estimates represent the change in RBC folate concentrations for each doubling in chemical concentrations. Blue illustrates a positive estimate and red illustrates a negative estimate.

**Figure 5:**
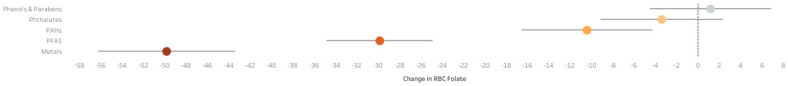
Pooled estimates and 95% CIs for the associations between environmental chemical mixtures and RBC folate using quantile g computation (Q-gcomp) analysis of adults from the NHANES database from 2007 to 2016 using 70 imputed datasets. Adjusted for age, race, family income-to-poverty ratio, education, NHANES cycle, smoking status, and diet. Additional adjustments for urinary creatinine were made for models with chemicals collected in urine. Estimates represent the association between a one quantile increase in the exposure family and a ng/mL change in RBC folate. Blue illustrates a positive estimate and red illustrates a negative estimate.

The ExWAS sensitivity analysis using imputed datasets revealed 21 chemicals that were statistically significantly negatively associated with RBC folate and one chemical that was statistically significantly positively associated with RBC folate (Figure 4). For instance, a doubling of cotinine (β = −31.1; 95%CI: −34.0, −28.2) was associated with a statistically significant decrease in RBC folate, and manganese (β = 4.8; 95%CI: 1.2, 8.4) was associated with a statistically significant increase in RBC folate after adjustment. The Q-gcomp sensitivity analysis using imputed datasets revealed that one quartile increases in PFAS (β = −29.9; 95%CI: −34.8, −24.9), metal (β = −49.8; 95%CI: −56.2, −43.4), and PAH (β = −10.4; 95%CI: −16.6, −4.3) mixtures were statistically significantly associated with decreases in RBC folate after adjustment (Figure 5). No other chemicals or chemical families were statistically significantly associated with changes in RBC folate after adjustment.

## 6. Discussion

In the present study, we used two statistical approaches, ExWAS and Q-gcomp, to evaluate the associations of 39 individual chemicals and 6 chemical mixtures with RBC folate concentrations in a sample of U.S. adults from NHANES (2007–2016). We found that several chemicals, including cadmium, lead, mercury, arsenic, PFDA, PFNA, PFUA, PFOA, PFOS, two hydroxynapthalene, three hydroxyfluorene, and cotinine were associated with lower RBC folate levels and MECPP, MBzP, manganese and selenium were associated with higher RBC folate levels. The ExWAS and complimentary Q-gcomp analysis yielded convergent results.

Althoμgh there are limited studies examining the associations between environmental chemicals and RBC folate concentrations, our findings are consistent with previous findings sμggesting an inverse association between lead and folate among lead-exposed workers and smoking and folate among Inuit women of childbearing age [30,31]. Additionally, one cross-sectional study has examined the associations among PFASs, and a PFAS mixture and red blood cell folate using Bayesian kernel machine regression (BKMR) and Q-gcomp, and found comparable results using data from NHANES 2007–2010 [32].

The potential pathways by which chemicals may affect folate levels are unclear; however, a few experimental studies sμggest possible biological mechanisms for the inverse associations between these environmental chemicals and RBC folate. For instance, PFASs have been shown to impact thyroid hormone levels [33,34] and thyroid stimulating hormones stimulate folate-dependent bioprocesses [35]. On the other hand, vitamin B complexes with folate have been shown to reduce cadmium and lead levels in rats, possibly by preventing absorption or facilitating increased excretion [36]. Also, mercury and PAHs are detoxified throμgh the glutathione detoxification system which requires folate for the metabolic processing of cysteine, a precursor of glutathione [37,38,39]. Finally, PAHs, PFASs, lead, and cadmium, have all been associated with increased homocysteine (Hcy) concentrations [40,41,42]. Hcy is inversely associated with bioavailable folate due to folate’s use in converting Hcy into either methionine or cysteine during one-carbon metabolism, and folate deficiency can cause Hcy buildup [43].

Regarding the observed positive associations, selenium has been shown to enhance concentrations of glutathione and would help to explain the positive associations between selenium and RBC folate observed in our study [39].

This study has several key limitations. First, the design of the study is cross-sectional with only one measurement representing exposure status available. This precludes making any causal claims, as the temporality of the relationship between environmental chemicals and folate is unclear. Given the use of biomarkers for measurements of environmental chemicals, we cannot exclude the potential for reverse causation, whereas folate levels impact measured biomarkers of exposure, and their metabolism. For instance, we cannot state whether these chemicals decrease the levels of folate or whether folate helps decrease the body’s chemical burden, or impacts how folate is transported or metabolized in target tissues. The relationship may also be due to unmeasured confounders that may impact both exposure and RBC folate. For example, previous studies have shown that folate and PFASs share transport carriers [44]. Second, because there were NHANES cycles that did not include certain chemicals, there were periods where some chemicals were missing from the analysis, especially for PAHs in the last included NHANES cycle (2015–2016). These gap may introduce selection bias; however, results from analyses run with an imputed dataset were similar to the results in our main analysis. Also, the gaps in the data are largely due to the NHANES cycles and the subset of the participants that was representative of NHANES participants as a whole, and most likely were not related to the other variables in our analysis.

The strengths of this study include the breadth of environmental chemicals available throμgh the NHANES dataset and the adoption of modern statistical approaches to analyze both mixtures and individual associations between environmental chemicals and RBC folate. Additionally, the large sample size and representativeness for the United States population provide increased generalization to non-institutionalized United States citizens, and adequate power for sensitivity analyses. Also, red blood cell folate is a long-term biomarker of folate status and is more resistant to acute changes in folate status and supplementation. Finally, we also adjusted for the HEI which is a good indicator of potential confounding by dietary patterns.

## 7. Conclusions

Our results sμggest significant relationships between exposures to some environmental chemicals and RBC folate, and these findings may have implications for public health. First, a ubiquitous and continuous source of chemical exposures that has a modest inverse association with folate can have important implications for public health given the role of folate in many biological processes. Second, vulnerable populations with insufficient folate levels are further burdened by preventable modifiable environmental factors that reduce folate levels.

This study, to our knowledge, is the first to examine the associations between mixtures of metals, PAHs, phenols and parabens, and phthalates with RBC folate levels. Our study’s results should be interpreted cautiously given the cross-sectional design. Future prospective studies can expand these preliminary findings by investigating temporally aligned exposures to environmental chemicals, folate levels, and potential health outcomes at later time points.

## Figures and Tables

**Table 1:** Mean concentrations of red blood cell (RBC) folate according to participants characteristics (NHANES; 2007–2016). Abbreviations: standard deviation (SD); family income-to-poverty ratio (FPR); healthy eating index (HEI). The *p*-Value calculation for Overall RBC Folate is not applicable (N/A).

	N	Mean	SD	*p*-Value
Overall RBC Folate	27,938	515.7	243.7	N/A
Sex				<0.001
Male	13,599	500.1	232.5	-
Female	14,339	530.6	252.9	<0.001
Age				<0.001
18 to 34	8022	439.1	172.1	-
35 to 64	13,650	506.3	220.4	<0.001
65 and Older	6266	634.4	315.0	<0.001
Race				<0.001
Mexican American	4441	478.4	199.2	-
Other Hispanic	3012	483.1	200.7	0.918
Non-Hispanic White	11,605	584.4	273.4	<0.001
Non-Hispanic Black	5768	440.6	210.3	<0.001
Other	3112	483.9	211.4	0.861
Education				<0.001
Less than 9th Grade	3107	489.8	236.0	-
9th to 11th Grade	4094	495.5	242.6	0.921
High School Graduate/GED	6309	514.4	255.4	<0.001
Some College or AA	8074	519.6	246.0	<0.001
College Graduate or Above	6254	539.0	229.1	<0.001
Missing	100	473.0	301.1	0.984
Smoking Status				<0.001
Has not Smoked in Last 30 Days	22,200	533.6	250.6	-
Smoked in Last 30 Days	5729	446.7	200.6	0.540
Missing	9	448.5	134.0	0.999
FPR				<0.001
Lowest Quartile	6335	475.5	223.7	-
2nd Quartile	6349	516.3	251.2	<0.001
3rd Quartile	6365	528.9	242.9	<0.001
Highest Quartile	6401	547.5	244.1	<0.001
Missing	2488	501.3	258.4	<0.001
Urinary Creatinine				<0.001
Lowest Quartile	6376	548.9	258.1	-
2nd Quartile	6372	531.8	253.3	<0.001
3rd Quartile	6473	500.0	225.0	<0.001
Highest Quartile	6469	471.4	210.1	<0.001
Missing	2248	549.4	288.3	0.999
Cycle				0.251
2007–2008	5600	538.0	265.0	-
2009–2010	6019	496.2	232.8	<0.001
2011–2012	5269	486.0	224.5	<0.001
2013–2014	5636	533.7	247.4	0.88
2015–2016	5414	524.7	242.1	0.03
HEI				<0.001
Lowest Quartile	6534	474.7	229.2	-
2nd Quartile	6533	498.7	237.6	<0.001
3rd Quartile	6534	528.8	251.1	<0.001
Highest Quartile	6534	565.4	249.5	<0.001
Missing	1803	499.0	232.5	<0.001

**Table 2:** Concentrations of environmental chemicals among adult NHANES participants from 2007 to 2016. Abbreviations: geometric mean (GM); geometric standard deviation (GSD); interquartile range (IQR); limit of detection (LOD).

Exposure	N	% < LOD	GM	GSD	IQR
Phthalates (ng/mL)					
Mono(carboxyisoctyl) phthalate (MCOP)	9012	1.22%	11.34	4.17	23.98
Mono-2-ethyl-5-carboxypentyl phthalate (MECPP)	9012	0.23%	15.19	3.25	22.90
Mono-n-butyl phthalate (MBP)	9012	2.24%	11.83	3.44	19.94
Mono-(3-carboxypropyl) phthalate (MCPP)	9012	9.55%	2.10	3.67	3.70
Mono-ethyl phthalate (MEP) (ng/mL)	9012	0.13%	61.78	4.94	153.48
Mono-(2-ethyl-5-hydroxyhexyl) phthalate (MEHHP)	9012	0.67%	9.68	3.51	15.90
Mono-(2-ethyl)-hexyl phthalate (MEHP)	9012	33.60%	1.61	3.10	2.63
Mono-isobutyl phthalate (MiBP)	9012	2.00%	7.49	3.12	12.00
Mono-isononyl phthalate (MNP)	9012	67.63%	1.05	2.74	0.61
Mono-(2-ethyl-5-oxohexyl) phthalate (MEOHP)	9012	0.95%	5.96	3.40	9.45
Mono-benzyl phthalate (MBzP)	9012	2.66%	4.88	3.72	9.80
Metals (µg/L)					
Cadmium (µg/L)	22,401	15.71%	0.36	2.24	0.38
Lead	22,401	0.29%	12.0	20.1	11.4
Manganese	10,787	0.00%	9.38	1.43	4.31
Mercury	22,401	9.85%	0.90	2.69	1.22
Selenium	10,787	0.00%	192.83	1.14	29.97
Total arsenic	9170	0.87%	8.35	3.17	12.40
PFAS (ng/mL)					
Perfluorodecanoic acid (PFDeA)	8963	16.82%	0.23	2.25	0.26
Perfluorohexane sulfonic acid (PFHxS)	8963	1.91%	1.39	2.61	1.79
2-(N-methylperfluoroctanesulfonamido)acetic acid (Me-PFOSA-AcOH)	8963	53.33%	0.16	2.52	0.23
Perfluorononanoic acid (PFNA)	8963	1.53%	0.91	2.11	0.88
Perfluoroundecanoic acid (PFUA)	8963	59.88%	0.15	2.27	0.13
Perfluorooctanoic acid (PFOA)	8961	0.22%	2.34	2.14	2.40
Perfluorooctane sulfonic acid (PFOS)	8961	0.39%	7.49	2.61	9.59
Phenols and parabens (ng/mL)					
Bisphenol A (BPA)	9013	0.65%	1.53	3.03	2.40
Triclosan	9013	7.51%	10.93	7.05	37.47
Butyl paraben	9013	0.18%	0.25	5.14	0.33
Methyl paraben	9013	2.49%	59.09	6.03	213.80
Propyl paraben (ng/mL)	9013	0.18%	7.33	9.78	43.70
PAHs (ng/L)					
One hydroxynaphthalene	7194	0.06%	2235.54	4.68	5480.00
Two hydroxynaphthalene	7221	0.00%	4474.30	3.20	8318.10
Three hydroxyfluorene	7256	1.86%	104.31	3.97	208.15
Two hydroxyfluorene	7267	0.00%	261.61	3.43	470.50
Three hydroxyphenanthrene	5439	1.84%	79.04	2.80	118.80
One hydroxyphenanthrene	7280	0.38%	123.95	2.56	164.73
Two hydroxyphenanthrene	5417	1.14%	68.29	2.55	90.00
One hydroxypyrene	7253	7.33%	118.89	2.84	176.30
Nine hydroxyfluorene	5448	0.00%	304.70	3.00	490.23
Cotinine (ng/mL)	27,646	27.41%	0.31	47.50	8.72

## Data Availability

All the data used in this manuscript are available at https://www.cdc.gov/nchs/nhanes/index.html. (accessed 1 December 2018).
